# Synthesis and Characterization of Polycaprolactone-Based Polyurethanes for the Fabrication of Elastic Guided Bone Regeneration Membrane

**DOI:** 10.1155/2018/3240571

**Published:** 2018-05-15

**Authors:** Shyh-Yuan Lee, Sheng-Chien Wu, Hsuan Chen, Lo-Lin Tsai, Jy-Jiunn Tzeng, Chih-Hsin Lin, Yuan-Min Lin

**Affiliations:** ^1^Department of Dentistry, National Yang-Ming University, No. 155, Sec. 2, Linong St., Beitou District, Taipei 112, Taiwan; ^2^Department of Stomatology, Veterans General Hospital Taipei, No. 201, Section 2, Shipai Road, Beitou District, Taipei City 112, Taiwan; ^3^Department of Dentistry, Taipei City Hospital, No. 145, Zhengzhou Rd., Datong District, Taipei 103, Taiwan; ^4^School of Dentistry, Taipei Medical University, No. 250 Wu-Xing Street, Taipei 110, Taiwan

## Abstract

The aim of this research is to synthesize polycaprolactone-based polyurethanes (PCL-based PUs) that can be further used for the fabrication of guided bone regeneration (GBR) membranes with higher tensile strength and elongation at break than collagen and PTFE membranes. The PCL-based PUs were prepared by the polymerization of polycaprolactone (PCL) diol with 1,6-hexamethylene diisocyanate (HDI) at different ratios using either polyethylene glycol (PEG) or ethylenediamine (EDA) as chain extenders. The chemical, mechanical, and thermal properties of the synthesized polymers were determined using NMR, FTIR, GPC, DSC, and tensile tester. The PCL and polyurethanes were fabricated as nanofiber membranes by electrospinning, and their mechanical properties and SEM morphology were also investigated.* In vitro* tests, including WST-1 assay, SEM of cells, and phalloidin cytoskeleton staining, were also performed. It was shown that electrospun membranes made of PCL and PCL-HDI-PEG (2 : 3 : 1) possessed tensile strength of 19.84 MPa and 11.72 MPa and elongation at break of 627% and 362%, respectively. These numbers are equivalent or higher than most of the commercially available collagen and PTFE membrane. As a result, these membranes may have potential for future GBR applications.

## 1. Introduction

Bone resorption is one of the most common problems among old people. The resorption of the alveolar bone makes it difficult for dentist to make dentures or to place dental implants. Currently, the most effective approach to bone regeneration is to use guided bone regeneration (GBR) membranes combining bone grafting materials. A GBR membrane must be strong, stretchable, and ideally biodegradable. Currently, the commercial GBR membranes can be divided into three categories, including nonresorbable membranes, natural bioresorbable, and synthetic bioresorbable membrane. Nonresorbable membranes are composed mainly of high-density polytetrafluoroethylene (d-PTFE), which requires an additional surgery for its retrieval. Its hydrophobicity and nonporosity can cause the death of the flap overlaid on it. The commercial product TXT-200 (Osteogenics, TX, USA) has a tensile strength of around 4.3 MPa and elongation at break of 301% [1]. Most bioresorbable membranes are collagen-based membranes. They show fewer problems regarding cell responses and toxic substances. The limitations of them are the high cost of raw materials, poor definition of the sources, and the difficulties of controlling degradation and mechanical properties [2]. The commercially available collagen membranes have a wide range of tensile strength and elongation at break of around 3.4 MPa~11.4 MPa and 9.6~46.8%, respectively [1, 3, 4]. Therefore, they may break during operation if care was not taken.

The aim of this research is to synthesize polycaprolactone-based polyurethanes (PCL-based PUs) that can be further used for the fabrication of guided bone regeneration (GBR) membranes higher tensile strength and elongation at break than collagen and PTFE membranes. Polyurethanes (PU) were utilized broadly in biomedical field, including cardiovascular [5], musculoskeletal [6, 7], and nerve regenerative [8, 9] applications. The use of polyurethanes in guiding bone regeneration membrane was not frequently reported. Dong et al. prepared GBR membranes made of polyurethane and hydroxyapatite [10]. However, the membranes were not porous, which is not beneficial to cell attachments. Recently, polyurethane membranes made of PCL, PDMS, and bismuth-doped nanohydroxyapatites were prepared [11]. The incorporation of the PDMS segment into the polymer backbone, however, may cause long-term harm to the body.

PU can be prepared by reacting diisocyanates with polyols in the presence of catalysts. The chain extenders can also be added to adjust the mechanical or thermal properties of PU. In biomedical area, polyurethanes were usually used in the form of “segmented polyurethanes” which can be present as “P-(D(C-D)_m_-P)_n_.” P are polyols, D are diisocyanates, and C is the chain extender [12]. Generally, the polyols (HO-R-OH) terminated by hydroxyl groups are “soft segments” of polyurethanes because of lower glass transition temperatures. Diisocyanates (OCN-R-NCO), the “hard segments,” are relatively smaller molecules which can react and connect the polyols and the chain extenders. The chain extenders are molecules with hydroxyl or amine groups at the ends that can be added for adjusting the properties of polyurethanes. Hexamethylene diisocyanate (HDI) was chosen in this study for the hard segment. It is cheap for large number of manufacturing and aliphatic that may cause less toxicity. Also, polyurethane products made from HDI do have enough mechanical properties for membranes fabrication [13].

A polycaprolactone diol was a specialized PCL composed of two polycaprolactones with an ethylene glycol so that the two terminated hydroxyl groups can react with diisocyanates to produce polyurethanes. Polyurethanes made from PCL diols display excellent hydrolytic ability [14]. It is a hydrophobic and biodegradable polyester [15] which has a low melting point around 60°C and a glass transition temperature around −60°C. Polyethylene glycols (PEG) are one of the mostly used biocompatible modifiers in polymers for chemical and biological applications [16], such as the surface of biomedical implant and drug delivery system [17]. Polyurethanes made from PEG were expected to be more hydrophilic and friendly for cell proliferation without toxicity or degradation burdens [18]. Ethylenediamine (EDA) is terminated by two amines (R-NH_2_) that can produce poly (urea urethane) by reacted with diisocyanates. There were already some polyurethane products that used EDA to reach ideal elongations and strengths, such as the artificial ligament reconstruction [7]. Polyurethanes containing EDA chain extenders exhibited enhanced elasticity, elongation, and strength for drug delivery application [19].

Electrospinning is a technique for generating fibrous membranes that was broadly used in scaffolds for tissue engineering, drug delivery, and wound dressings. The main advantage of electrospinning is the ability to fabricating fibers with diameters from several micrometers to less than 100 nanometers [20]. Currently, electrospun nanofibers made of PCL and its copolymers are widely used in tissue engineering [21] and drug delivery [22] because of their biodegradability, good mechanical properties, and the approval of FDA. So far, the use of PCL-based PUs composed of PCL as soft segment, HDI as hard segment, and PEG or EDA as chain extender for electrospinning of GTR membrane has not been reported yet. Therefore, in this study, their potential in GBR membrane was evaluated.

## 2. Materials and Methods

### 2.1. Synthesis of the Polyurethanes

**Figure 1:**
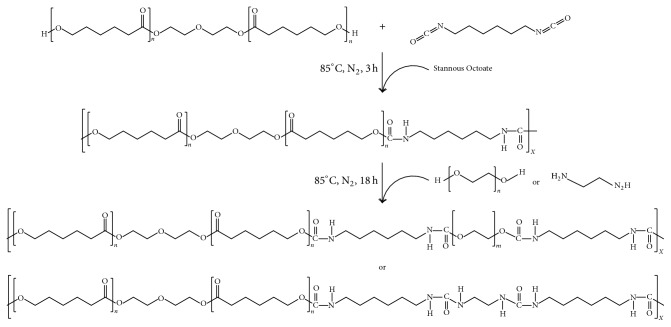
The two-step polymerization of polyurethanes.

The PCL-based polyurethanes were synthesized by a two-step solution polymerization method (Figure 1). Before the reaction, polycaprolactone diol (Acros, USA) with the molecular weights (MW) of 2000 dalton was dried under nitrogen atmosphere at 160°C for 1 h and the solvent toluene was dried over 4-Å molecular sieves (Alfa Aesar, England) overnight to remove the residual water. Hexamethylene diisocyanate (Acros, USA), polyethylene glycol (Showa, Japan) with the MW of 1000 dalton, ethylenediamine (Acros, USA), and the catalyst stannous octoate were used as received. In the first step, a solution of PCL diol in toluene was added dropwise to a quick-stirred solution of HDI in toluene at 60°C under a dry nitrogen atmosphere. Meanwhile, stannous octoate (Sigma-Aldrich, USA) was added to the system directly at the ratio of 0.1% v/v to the solvent. The reaction was carried out at 85°C for 3 h. The stoichiometric ratios of PCL diol to HDI were shown in Table 1. In the second step, the triblock polyurethanes were produced by the reaction of prepolymers with chain extenders PEG or EDA. The sample PCL-HDI (1 : 1), which contained HDI and PCL at 1 : 1, did not undergo this step. The solution of PEG or EDA in toluene was prepared and added dropwise into the stirred prepolymer solution. The stoichiometric ratios of the prepolymers to chain extenders were shown in Table 1. The polymerization was kept at 85°C for more than 18 h. After the completion of the reaction, more than five-time-volume hexane was added to the polymer solution quickly at 0°C to precipitate the polymers. The whole solution was then filtered through 6 *μ*m filter paper to retain the polymers. The precipitation steps were repeated for three times. Finally, the polymers were dried under vacuum at 50°C for 2 days.

### 2.2. Characterizations of the PCL-Based Polyurethane Products

Before NMR measurement, 10 mg of PCL-based polyurethane was dissolved in 1 ml of deuterated chloroform (Seedchem, Australia) with tetramethylsilane as an internal standard. ^1^H-NMR spectra were carried out at a temperature of 300 K using Bruker Ascend 400 MHz Nuclear magnetic resonance spectrometer in our experiment. Each specimen was scanned 32 times to obtain the average of spectrum. Chemical shifts were referenced relatively to chloroform at 7.26 ppm in ^1^H-NMR spectra. Peaks on those spectra were delimited and integrated using the Topspin 3.0 software.

### 2.3. Characterizations of the Chemical Structures of the PCL-Based Polyurethane Using Fourier Transform Infrared Spectrometry

A NICOLET iS5 Fourier transform infrared (FTIR) spectrometer (Thermo Scientific, Massachusetts, USA) equipped with ATR (Attenuated total reflectance) ZnSe crystal was used to characterize the chemical bonding of the PCL-based polyurethane. Every sample was taken for sixteen scans and the spectra were ranged from 400 to 4000 cm^−1^.

### 2.4. Measurement of Molecular Weight Using Gel Permeation Chromatography

The molecular weights of the PCL-based PU products were determined by an organic-solvent modular gel permeation chromatography (GPC) system (Waters, USA). The calibration was carried out with polystyrene molecular weight standards. The samples were dissolved in THF (tetrahydrofuran) at 0.2% w/v and then filtered through a 0.2 *μ*m filter before injecting to the system. Approximately 2 ml solutions of each sample were used.

### 2.5. Evaluation of the Thermal Properties of PCL-Based Polyurethane Using Differential Scanning Calorimetry

A TA Q100 differential scanning calorimeter (DSC) was used to measure the glass transition and melting temperature (*T*_*g*_ and *T*_*m*_) of the PCL-based PU. 3–5 mg of the samples was scanned at a heating rate of 10°C/min under dry nitrogen flow. The sample was first cooled down to −85°C and then heated to 150°C. The thermocycle was repeated twice and the second cycle was recorded in order to make sure that the polymers were recrystallized evenly.

### 2.6. Fabrication of the PCL and PCL-Based Polyurethane Membranes

PCL and PCL-based PU membranes were prepared using solvent casting and electrospinning methods. For solvent casting, 5% polymers/chloroform were poured into glass Petri dishes and covered by aluminum foils with holes in order to allow slow solvent evaporation. For electrospinning, 10–30% (w/v) of polymers in chloroform were injected from a plastic syringe at the feeding rate 0.005–0.033 ml/min through a stainless needle (21–27 gauge), on which a high voltage of 5–25 kV was applied. The collector was placed 5–25 cm away from the need tip. The electrospinning procedure was performed at room temperature.

### 2.7. Characterizations of the PCL and PU Membranes

A universal testing machine (Cometech QC-513B1, Taiwan) equipped with a 10 kg load cell was used to measure the tensile strength, the elongations at break, and Young's modulus of the membranes. The samples were cut into about 20 mm × 5 mm × 0.3 mm and the testing speed was 200 mm/min. All the measurements, including the test of solvent-casted and electrospun membranes, were repeated for at least three times.

### 2.8. Recording the Nanofiber Membrane Structures Using Scanning Electron Microscopy

The fiber diameter and distribution, the pore sizes, and overall topography of the electrospun membranes were recorded using scanning electronic microscopy (SEM). All samples were coated with a layer of gold on the surface. The electrospun membranes photographs were taken using a JOEL JSM-7600F SEM at an accelerating voltage of 5 kV. The fiber diameters were measured using ImageJ software.

### 2.9. Culture of MG-63 Cells

The culture medium of MG63 osteosarcoma cells was composed of Dulbecco's modified eagle medium (DMEM) supplemented with 10% fetal bovine serum (FBS) and 1% pen-strep-amp antibiotics. Cells were cultured in 100 mm Petri dishes with the replacement of culture medium every other day and kept in a humidified incubator at 37°C in the presence of 5% CO_2_.

### 2.10. Proliferation Test of MG63 Cells Using WST-1 Assay

The cytotoxicity of PCL and PU membranes on MG63 was performed using WST-1 cell proliferation assay. MG63 cells were seeded in 24-well plates at the density of 2 × 10^−4^ cells per well and maintained in the incubator at 37°C under 5% CO_2_ atmosphere. The WST-1 assay was conducted 1, 4, and 7 days after seeding according to the manufacturer's protocols.

### 2.11. Recording of Cell Morphology by SEM

The MG63 cell morphology on electrospun membrane was recorded using SEM. MG63 cells were cultured at the density of 5 × 10^−4^ cells per well on the membranes glued at the bottom of 24-well plates. After 24 h, cells were washed with PBS, fixed with 4% paraformaldehyde in PBS for 15 min, and then washed with PBS for three times. The samples were immediately dehydrated in a series of aqueous ethanol solutions at the concentrations of 50%, 70%, and 80% for 10 min and then 95% and 100% for 15 min. Following the ethanol dehydration, the samples were put into a freeze dryer overnight to remove the residual ethanol. All samples were coated with a layer of gold on the surface, and their scanning electron micrograph images were taken at an accelerating voltage of 5 kV using a JEOL JSM-7600F SEM (JOEL, USA).

### 2.12. Cytoskeleton and Nucleus Staining of MG63 Cells by Rhodamine Phalloidin and DAPI

MG63 cells were seeded at the density of 5 × 10^−4^ cells per well on every electrospun membrane, which was glued at the bottom of 24-well plates. After 24 h incubation, MG63 cells were washed with PBS, fixed with 4% paraformaldehyde in PBS for 15 min, and rinsed with 0.1% Triton™ X-100 (Thermo, USA) in PBS for 1 min for permeabilization. After several washes with PBS, the rhodamine phalloidin (Invitrogen, USA) at 1 : 40 in PBS was added to the fixed cells (0.5 ml per well) for 30 min for fluorescence staining. After several washes, cells were stained with DAPI (4′,6-diamidino-2-phenylindole, Invitrogen, USA) and mounted with coverslips. The fluorescence images were collected using fluorescence microscope.

### 2.13. Statistical Analysis

The results of tensile tests and WST-1 assays and fiber diameters were expressed as mean ± standard error of the mean. Comparative studies of means were analyzed using one-way ANOVA with a statistical significance at *p* < 0.05.

## 3. Results

### 3.1. Analysis of the Structure and Properties of the Polymers

**Figure 2:**
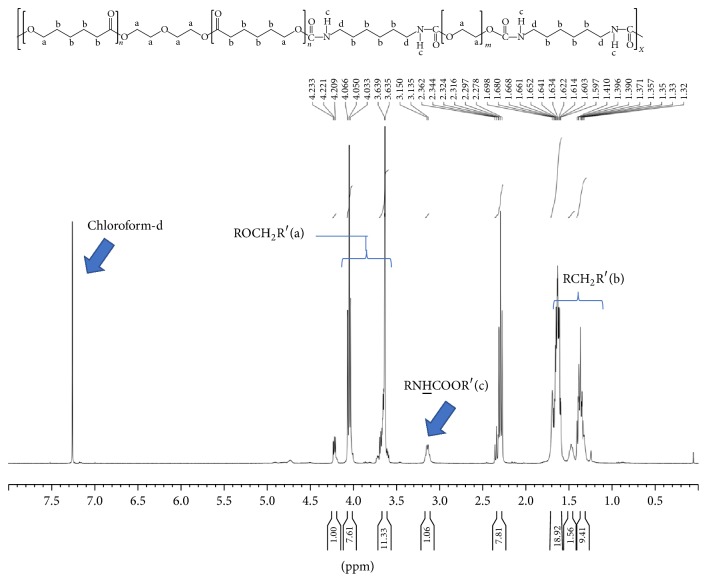
The NMR spectrum of PCL-HDI-PEG (2 : 3 : 1).

**Figure 3:**
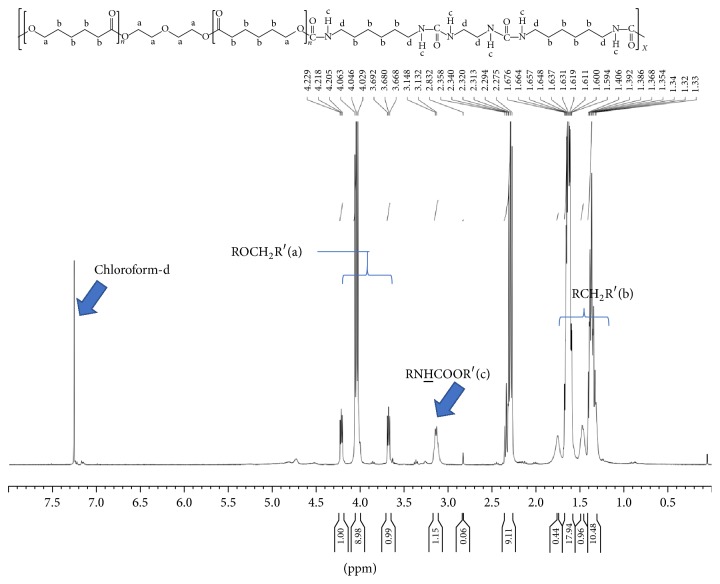
The NMR spectrum of PCL-HDI-EDA (2 : 3 : 1).

NMR spectrometer was performed to evaluate the chemical structures of the PCL-based PU by detecting ^1^H (Table 2). Because three PCL-HDI-PEG or PCL-HDI-EDA materials have similar peaks, only the spectra of PCL-HDI-PEG (2 : 3 : 1) and PCL-HDI-EDA (2 : 3 : 1) were shown (Figures 2 and 3). The NMR spectra of the polymers showed three areas of characteristic peaks at 3.15 ppm (RNHCOOR, c), 3.3–4 ppm (ROCH_2_R, b), and 1.2–1.6 ppm (RCH_2_R, a) [23–25]. The peak at 3.15 ppm stood for the presence ratio of the urethane groups. The proton ratios of the PCL-based PU were calculated and showed in Table 2 as (RNHCOOR′ : ROCH_2_R′ : RCH_2_R′). Experimental ^1^H ratio of most samples matched the theoretical ratio pretty well. However, PCL-HDI-PEG (1 : 2 : 1), PCL-HDI-EDA (2 : 3 : 1), and PCL-HDI-EDA (1 : 3 : 2) showed discrepancy between experimental ^1^H ratio and theoretical ^1^H ratio.

### 3.2. Fourier Transform Infrared Spectra of the PCL-Based PU

**Figure 4:**
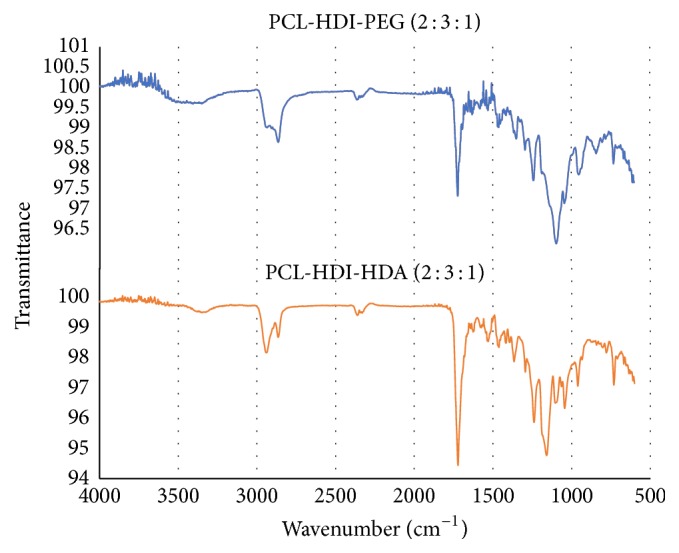
The FTIR spectra of (a) PCL-HDI-PEG (2 : 3 : 1) and (b) PCL-HDI-EDA (2 : 3 : 1).

All PCL-based PUs were characterized using the FTIR spectrometer because three PCL-HDI-PEG or PCL-HDI-EDA materials have similar peaks; only PCL-HDI-PEG (2 : 3 : 1) (Figure 4(a)) and PCL-HDI-EDA (2 : 3 : 1) (Figure 4(b)) were shown. Many characteristic peaks including bending C-H bonds at 800–1465 cm^−1^, C=O bonds at 1650–1818 cm^−1^, and stretching C-H bonds at 2800–3000 cm^−1^ can be found. In both spectra, the characteristic peak of isocyanates (N=C=O) at 2275–2400 cm^−1^ did not appear, suggesting that HDI was totally consumed during polymerization.

### 3.3. Molecular Weights of the PCL-Based PU

The *M*_*n*_ (number average molecular weight), *M*_*w*_ (weight average molecular weight), and *M*_*p*_ (peak average molecular weight) of the PCL-based PU were listed in Table 3. Meanwhile, the polydispersity, indicating the dispersed degrees of the molecular weights, calculated by *M*_*w*_/*M*_*n*_, shows the GPC result of the products. The polydispersity of PCL-based PU ranges within 1.43~2.27.

### 3.4. Thermal Properties of the PCL-Based PU

Thermal properties of the PCL-based PU were evaluated using differential scanning calorimeter. The result was listed in Table 4. *T*_*g*_ of all the polyurethane products ranged from −47 to −56°C. Sample PCL-HDI-EDA (2 : 3 : 1) had the highest *T*_*g*_ of −47.5°C. The *T*_*m*_ of the PCL-based PU ranges within 27~43°C. PCL-HDI-EDA (1 : 3 : 2) had the lowest *T*_*m*_ of 27.94°C.

### 3.5. Fibrous Structures of the Electrospun Membranes

**Figure 5:**
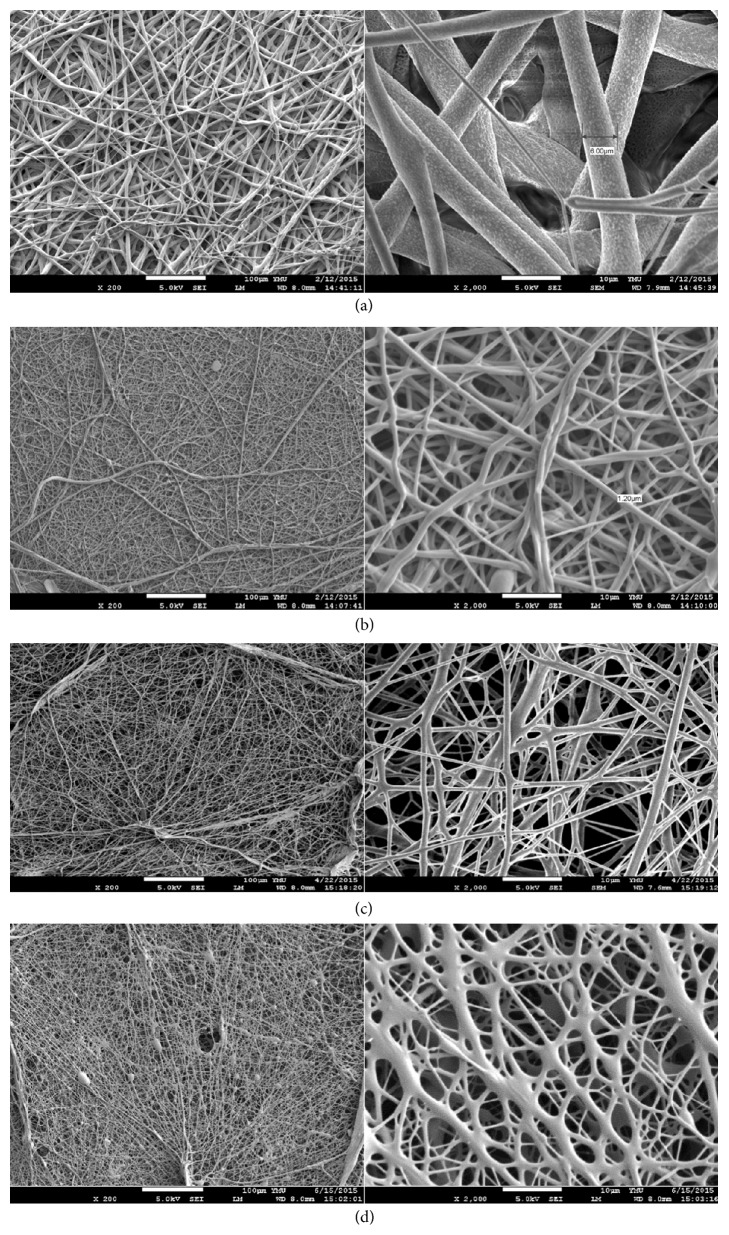
From (a) to (d): SEM images of electrospun membranes made of PCL, PCL-HDI-PEG (1 : 2 : 1), PCL-HDI-PEG (2 : 3 : 1), and PCL-HDI-EDA (2 : 3 : 1) at 200x (left column) and 2000x magnifications (right column).

Table 5 showed the best electrospun conditions of three PCL-based PUs. Other three PCL-based PUs cannot form a membrane using electrospinning with similar electrospinning conditions; therefore, they were not listed in the table. The fiber diameter of PCL membrane is 6.9 *μ*m (Figure 5, Table 7). The membrane had a layer by layer structure with majority of thick fibers covered by very few thin fibers. The electrospun PCL-HDI-PEG (1 : 2 : 1) membranes had a dense and evenly distributed fibrous structure. The fiber diameter is 1.05 *μ*m (Figure 5, Table 7). The electrospun PCL-HDI-PEG (2 : 3 : 1) membranes had fibrous structures similar to PCL-HDI-PEG (1 : 2 : 1). In contrast, the electrospun PCL-HDI-EDA (2 : 3 : 1) membranes had a poorly defined and nonhomogenous fibrous structure, very likely due to the dissolution of the unevaporated solvent during the electrospinning process. The fiber diameter of electrospun PCL is significantly larger than all PCL-based PUs (Table 7). There is no statistical difference between each PCL-based PU.

### 3.6. Tensile Properties of the Solvent-Cased and Electrospun Membranes

Mechanical properties of all the solvent-casted and electrospun membranes were listed in Tables 6 and 7. Some PCL-based PUs showed very low solubility in the solvent, so they failed to form membranes using solvent casting and electrospinning, including PCL-HDI-EDA (1 : 2 : 1) and PCL-HDI-EDA (1 : 3 : 2). In Table 6, it can be found that the solvent-casted PCL membranes showed the highest tensile strength and modulus of elasticity. It was worth noticing that solvent-casted PCL-HDI-EDA (2 : 3 : 1) had higher tensile strengths and higher modulus of elasticity than solvent-casted PCL-HDI-PEG (2 : 3 : 1), it is because of different types of chain extenders. The strength, modulus, and elongation of the electrospun membranes were much lower than their solvent-casted counterparts, except for the electrospun PCL, which has similar elongation at break as the solvent-casted PCL. Electrospun membranes made of PCL and PCL-HDI-PEG (2 : 3 : 1) possessed tensile strength of 19.84 MPa and 11.72 MPa and elongation at break of 627% and 362%, respectively. These numbers are equivalent or higher than most the commercially available collagen and PTFE membrane.

### 3.7. Cell Responses to the PCL and Polyurethane Membranes

**Figure 6:**
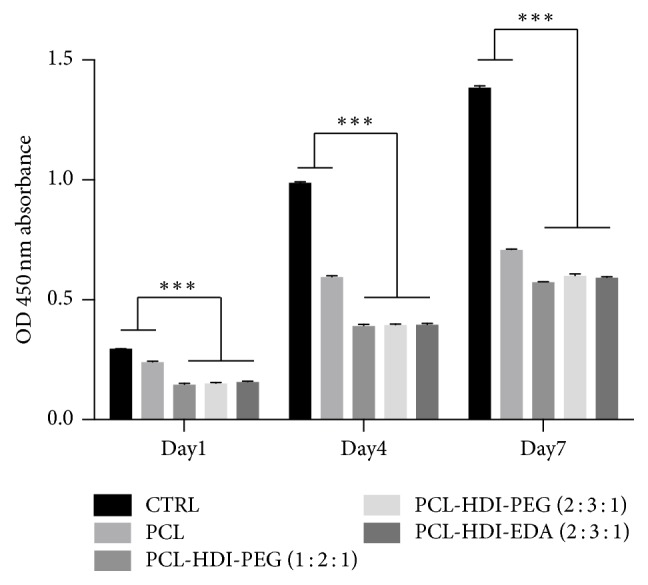
The results of cell proliferation of MG63 cells on electrospun membranes using WST-1 assay. Asterisks *∗∗∗* stands for the statistically significant difference with *P* < 0.001.

Figure 6 shows the cell proliferation on control and four different electrospun membranes on 1, 4, and 7 days. The control is the 24-well plates. Cell proliferation on control group and PCL was significantly better than other PCL-based PU membranes on days 1, 4, and 7. The three PU membranes had no significant difference with each other.

### 3.8. Scanning Electron Micrographs of MG63 Cells Grown on Electrospun Membranes

**Figure 7:**
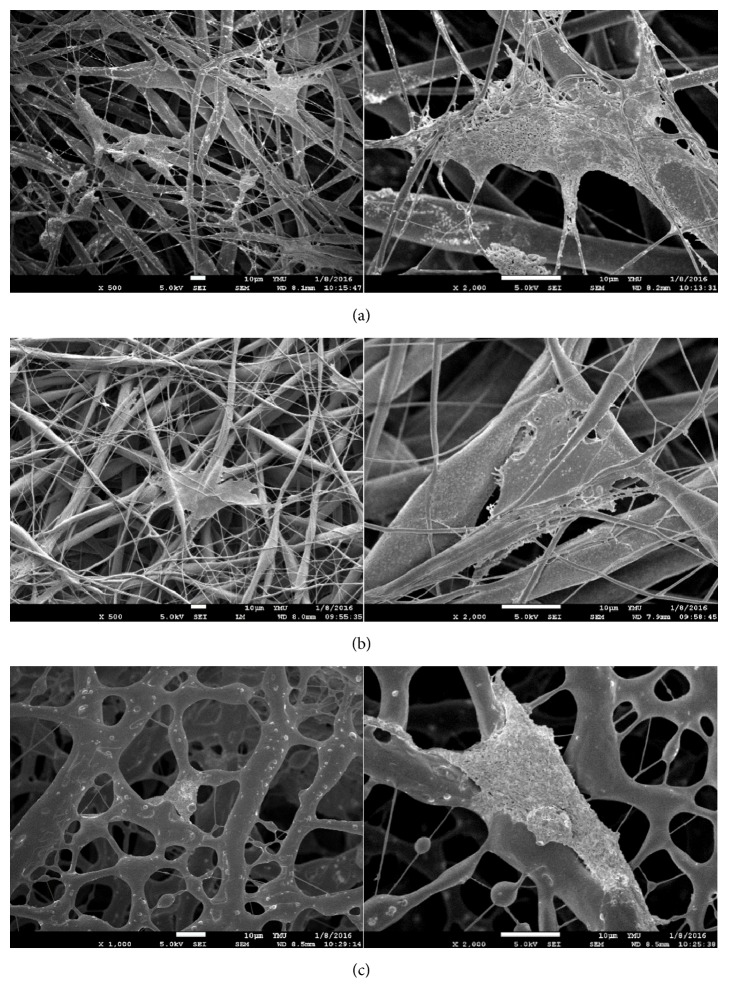
(a) to (c) are the scanning electron micrographs of the MG63 cells on electrospun membranes made of different materials, including PCL, PCL-HDI- PEG (2 : 3 : 1), and PCL-HDI- EDA (2 : 3 : 1).

The morphologies of cells were observed using SEM after the ethanol serial dehydration and freeze-drying. Electrospun PCL-HDI-PEG (1 : 2 : 1) membranes were too fragile and hydrophilic to be used in this characterization.  Figure 7 showed the morphology of MG63 cells cultured for 24 h on electrospun membranes. Cells on PCL and PCL-HDI-PEG (2 : 3 : 1) membranes were more stretched out than on PCL-HDI-EDA (2 : 3 : 1). The cell density on PCL membranes was higher than other two electrospun membranes.

### 3.9. Cytoskeleton and Nucleus Staining of Cells

**Figure 8:**
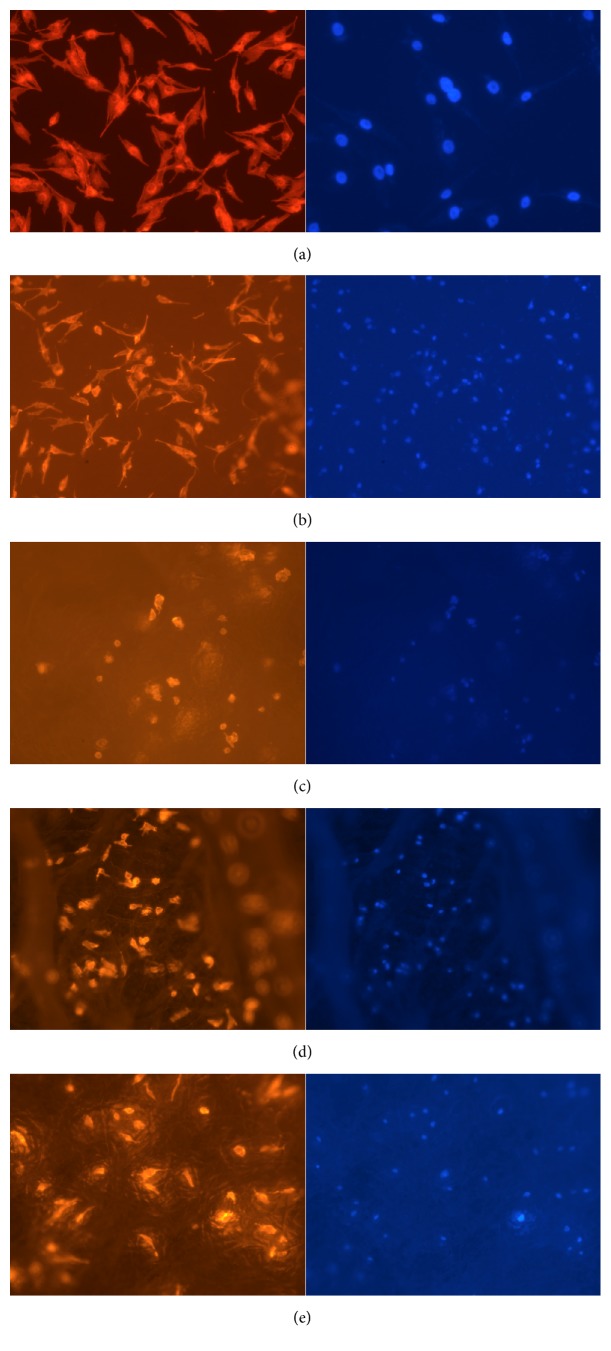
(a) to (e) are fluorescent images of phalloidin and DAPI staining of MG63 cells cultures on different materials, including 24-well plate (Control group), electrospun PCL membranes, electrospun PCL-HDI-PEG (1 : 2 : 1) membranes, electrospun PCL-HDI-PEG (2 : 3 : 1) membranes, and electrospun PCL-HDI-EDA (2 : 3 : 1) membranes.

The cytoskeleton and shape of MG63 cells on 24-well plates and electrospun membranes were investigated by staining the cell with rhodamine phalloidin and DAPI (Figure 8). Cells cultured on 24-well plates exhibited most clear cytoskeleton structures and spindle-like shapes, followed by electrospun PCL, PCL-HDI-EDA (2 : 3 : 1), and PCL-HDI-PEG (2 : 3 : 1). In contrast, cells on PCL-HDI-PEG (1 : 2 : 1) membranes expressed very weak cytoskeleton formation.

## 4. Discussion

In this research, seven different PCL-based PUs were synthesized by additional polymerization of PCL diol, HDI, and chain extender PEG or EDA at different ratios; NMR and FTIR were then employed to evaluate the chemical structures of PCL-based PUs. The protons of PCL-based PU can be categorized into 3 different groups, including peaks at 3.15 ppm (RNHCOOR), 3.3–4 ppm (ROCH_2_R), and 1.2–1.6 ppm (RCH_2_R), and the corresponding ^1^H peaks were found in the NMR spectra. The areas under these curves were proportional to the percentage of every characteristic ^1^H group among all protons. All PCL-based PUs showed ^1^H ratios close to the theoretical ratios at Table 2, except PCL-HDI-PEG (1 : 2 : 1), PCL-HDI-EDA (2 : 3 : 1), and PCL-HDI-EDA (1 : 3 : 2). This outcome might be caused by the self-polymerization of isocyanates of HDI. The most common products created by this reaction were biuret linkages [26, 27]. The biuret linkages created cross-linking between molecules and the ratios of protons in urethane groups (RNHCOOR′) were therefore higher than other ones. In order to prevent the biuret formation, the reaction time and temperature of the PU synthesis had to be shorter and lower [28]. Another way to prevent the biuret formation is to control the amount of the catalyst [28, 29]. In FTIR spectrum, it was worth noticing that isocyanate group (N=C=O), which had peaks at 2275–2400 cm^−1^, was not found in all PCL-based PUs. Because isocyanates were the characteristic groups of HDI, the absence of isocyanates in the final products proved that the raw materials of the PCL-based PU were consumed completely.

GPC results showed that our PCL-based PUs had lower molecular weights than commercial PCL, whose *M*_*w*_ was 80000. It might be one of the reasons that PCL-based PU had lower tensile strength and modulus of elasticity [30]. An ideal polydispersity index of polymers synthesized by step-growth polymerization is below 2.0 [31]. The polydispersity of PCL-based PU ranges within 1.43~2.27. The slight higher polydispersity index of some groups can be caused by the allophanate formation, which is the reaction of urethanes and the adjacent isocyanate groups, during polymerization. The allophanates result in the cross-linking of the growing polyurethane chain and thus a broader polydispersity index range.

DSC tests showed that the melting temperature of our PUs ranged from 27°C to 43°C. For the clinical applications, the melting temperature (*T*_*m*_) of the PCL-bases PUs must be higher than 37°C. However, only four PUs, including PCL-HDI (1 : 1), PCL-HDI-PEG (2 : 3 : 1), PCL-HDI-PEG (1 : 3 : 2), and PCL-HDI-EDA (2 : 3 : 1), met the requirement.

The fabrication of the electrospun membranes was carried out using various conditions, including concentrations of polymer solution, voltages imposed on the metal collector, distances between needle tips and the collector, needle gauge, and feeding rates. The commercial PCL was utilized as a control group in this section. In our experimental setup, high concentrations of the polymer solutions blocked the pipeline. While low concentrations of polymers were employed, the solvent chloroform did not evaporate during the flight to the collector. High voltages over 20 kV made the fibers spread in the space between the needle tips and the collector without attaching to the collector. In contrast, low voltages caused more visible beads in the membranes. The distances between needle tips and the collector should be precisely controlled. Otherwise, the fibers could also spread in the space between the needle tips and the collector without attaching to the collector. It was found that large needle (21 gauges) and low feeding rates (near the lowest limitation of electrospun unit, 0.005 ml/s) showed better results, especially for the PCL-based PU membranes.

The SEM images showed that electrospun PCL membranes had larger fiber diameters and more regular fiber distribution than PCL-based PUs. Cells cultured on PCL membranes also displayed the highest proliferation and vitality. It means that the fiber distribution has strong effects on cell growth [32, 33]. From Figure 5, it can be found that fibers of PCL group were the most evenly distributed, followed by PCL-HDI-PEG. PCL-HDI-EDA (2 : 3 : 1) membranes had fusions at the interactions, which were very likely caused by the dissolution of the polymer into the unevaporated solvent. This problem could be solved by the adjustment of concentrations or solvents.

Solvent-casted PCL-HDI-EDA (2 : 3 : 1) membranes exhibited much higher elongations and comparable tensile strength than solvent-casted PCL. Nevertheless, electrospun PCL-based PU membranes had significant reduction in tensile strengths, modulus of elasticity, and elongations compared to the electrospun PCL or their solvent-casted counterparts. According to Bottino and his coworkers [34], commercial GBR membranes have tensile strengths from 3.5 to 22.5 MPa. It indicated that the mechanical strengths of our electrospun membranes were not as strong as we predicted. In this case, the reasons why mechanical properties of electrospun PU membranes were reduced were worth discussing. Some researchers suggested that the high voltages will lead the polymer arrange in specific direction and display better mechanical properties [35]. Also, increasing the molecular weights of PCL-based PUs might be useful for the enhanced mechanical properties [30]. Despite so, electrospun PCL and PCL-HDI-PEG (2 : 3 : 1) possessed tensile strength of 19.84 MPa and 11.72 MPa and elongation at break of 627% and 362%, respectively. These numbers are equivalent or higher than most commercially available collagen and PTFE membrane.

In this study, MG63 human osteosarcoma cells were used for* in vitro* tests because it was broadly used as an* in vitro* model for evaluating the effects of many biomaterials [36, 37]. The cell proliferation tests were performed only on electrospun membranes because porous membranes were preferred for flap survival. The electrospun PCL group supported significantly better cell proliferation than other three PCL-based PUs membranes. It might be because its significantly larger and well-distributed fibers were suitable for cell proliferation [32, 33]. In SEM characterization, we found that cells on PCL membranes stretched out well, so did the cells on PCL-HDI-PEG (2 : 3 : 1) membranes. Cells on PCL-HDI-EDA (2 : 3 : 1) membranes did not stretch out and form spindle-like cells. Similar results can also be found in rhodamine phalloidin and DAPI staining. Because electrospun PCL and PCL-HDI-PEG (2 : 3 : 1) have better overall performance in terms of tensile strength, elongation at break, and cell responses, it was believed that they have higher potential in GBR applications.

## 5. Conclusion

In this study, several kinds of PCL-based PUs were successfully synthesized by the polymerization of PCL diol, HDI, and chain extenders PEG or EDA. Among all the electrospun membranes, PCL and PCL-HDI-PEG (2 : 3 : 1) were found to show the highest elongation at break, tensile strength, and* in vitro* cell responses; therefore, these two materials have the potential for the guided bone regeneration. In the future, more tests should be performed regarding their degradation behavior and* in vivo* performance.

## Figures and Tables

**Table 1:** The stoichiometry of the polyurethane materials.

Polymers	Materials			
	PCL diol	HDI	PEG	EDA
PCL-HDI (1 : 1)	1	1	0	0
PCL-HDI-PEG (2 : 3 : 1)	2	3	1	0
PCL-HDI-PEG (1 : 2 : 1)	1	2	1	0
PCL-HDI-PEG (1 : 3 : 2)	1	3	2	0
PCL-HDI-EDA (2 : 3 : 1)	2	3	0	1
PCL-HDI-EDA (1 : 2 : 1)	1	2	0	1
PCL-HDI-EDA (1 : 3 : 2)	1	3	0	2

**Table 2:** ^1^H ratio of the NMR spectra of polyurethane products. Asterisks *∗* stands for the statistically significant difference with *P* < 0.05.

Polymers	Proton (^1^H)	Theoretical ratio	Experimental ratio
PCL-HDI (1 : 1)	RNHCOOR′ : ROCH_2_R′ : RCH_2_R′ (c : b : a)	1 : 35 : 10	1 : 29 : 9
PCL-HDI-PEG (2 : 3 : 1)		1 : 24 : 17	1 : 27 : 18
PCL-HDI-PEG (1 : 2 : 1)		1 : 36 : 35	1 : 17 : 16^*∗*^
PCL-HDI-PEG (1 : 3 : 2)		1 : 13 : 22	1 : 11 : 18
PCL-HDI-EDA (2 : 3 : 1)		1 : 18 : 5	1 : 26 : 9^*∗*^
PCL-HDI-EDA (1 : 2 : 1)		1 : 19 : 5	1 : 19 : 5
PCL-HDI-EDA (1 : 3 : 2)		1 : 8 : 3	1 : 16 : 5^*∗*^

**Table 3:** The GPC results of the polyurethane products.

Polymers	GPC results			
	*M*_*n*_	*M*_*w*_	*M*_*p*_	Polydispersity
PCL-HDI (1 : 1)	49494	81087	80591	1.68313
PCL-HDI-PEG (2 : 3 : 1)	34076	69916	75849	2.05179
PCL-HDI-PEG (1 : 2 : 1)	50992	85794	82974	1.682495
PCL-HDI-PEG (1 : 3 : 2)	22695	41075	32815	1.809889
PCL-HDI-EDA (2 : 3 : 1)	41155	93716	102739	2.277146
PCL-HDI-EDA (1 : 2 : 1)	28866	63934	66155	2.214852
PCL-HDI-EDA (1 : 3 : 2)	13734	19750	18103	1.438019

**Table 4:** The DSC results of the polyurethane products.

Polymers	Thermal properties		
	*T*_*g*_ (°C)	*T*_*m*_ (°C)	Enthalpy of fusion (J/g)
PCL-HDI (1 : 1)	−54.69	43.76	44.39
PCL-HDI-PEG (2 : 3 : 1)	−55.32	39.59	54.05
PCL-HDI-PEG (1 : 2 : 1)	−53.30	35.26	44.78
PCL-HDI-PEG (1 : 3 : 2)	−56.64	42.01	44.91
PCL-HDI-EDA (2 : 3 : 1)	−47.49	43.64	26.70
PCL-HDI-EDA (1 : 2 : 1)	−51.69	34.61	21.62
PCL-HDI-EDA (1 : 3 : 2)	−51.74	27.94	19.75

**Table 5:** The condition of electrospinning.

	Concentration (W/v%)	Voltage (V)	Distance (cm)	Needle gauge	Feeding rate (ml/h)
PCL	13	13	10	23	2
PCL-HDI-PEG (1 : 2 : 1)	30	12	15	21	0.6
PCL-HDI-PEG (2 : 3 : 1)	20	13	10	21	0.3
PCL-HDI-EDA (2 : 3 : 1)	15	13	7.5	21	0.9

**Table 6:** The mechanical properties of solvent-casted membranes.

Solvent-casted membranes	Mechanical properties		
	Tensile strength	Modulus of elasticity	Elongation at break
	(MPa)	(MPa)	(%)
PCL	69.10	59.94	621.02
PCL-HDI (1 : 1)	30.78	40.92	1211.86
PCL-HDI-PEG (2 : 3 : 1)	33.60	40.78	1162.46
PCL-HDI-PEG (1 : 2 : 1)	28.17	35.01	788.83
PCL-HDI-PEG (1 : 3 : 2)	Too fragile to conduct tests		
PCL-HDI-EDA (2 : 3 : 1)	51.99	43.41	1553.00
PCL-HDI-EDA (1 : 2 : 1)	Poor solubility in solvents for solvent casting		
PCL-HDI-EDA (1 : 3 : 2)			

**Table 7:** The mechanical properties and fiber diameter of electrospun membranes. The fiber diameter of PCL is significantly larger than all PCL-based PUs. There is no statistical difference between each PCL-based PU.

Electrospun membranes	Mechanical properties			Fiber diameter (*μ*m)
	Tensile strength (MPa)	Modulus of elasticity (MPa)	Elongation at break (%)	
PCL	19.84	26.32	627.58	6.9 ± 0.8
PCL-HDI (1 : 1)	No membrane with fibrous structures was formed			
PCL-HDI-PEG (1 : 2 : 1)	5.39	10.59	214.59	1.05 ± 0.17
PCL-HDI-PEG (2 : 3 : 1)	11.72	15.31	362.14	0.86 ± 0.08
PCL-HDI-EDA (2 : 3 : 1)	5.53	11.34	241.22	1.09 ± 0.16

## References

[1] Ha Y.-Y., Park Y.-W., Kweon H., Jo Y.-Y., Kim S.-G. (2014). Comparison of the physical properties and in vivo bioactivities of silkworm-cocoon-derived silk membrane, collagen membrane, and polytetrafluoroethylene membrane for guided bone regeneration.

[2] Coïc M., Placet V., Jacquet E., Meyer C. (2010). Mechanical properties of collagen membranes used in guided bone regeneration: A comparative study of three models.

[3] Bozkurt A., Apel C., Sellhaus B. (2014). Differences in degradation behavior of two non-cross-linked collagen barrier membranes: An in vitro and in vivo study.

[4] Ortolani E., Quadrini F., Bellisario D., Santo L., Polimeni A., Santarsiero A. (2015). Mechanical qualification of collagen membranes used in dentistry.

[5] Nelson A. M., Long T. E. (2014). Synthesis, properties, and applications of ion-containing polyurethane segmented copolymers.

[6] Bi X., You Z., Gao J., Fan X., Wang Y. (2014). A functional polyester carrying free hydroxyl groups promotes the mineralization of osteoblast and human mesenchymal stem cell extracellular matrix.

[7] Gisselfält K., Edberg B., Flodin P. (2002). Synthesis and properties of degradable poly(urethane urea)s to be used for ligament reconstructions.

[8] Saad B., Hirt T. D., Welti M., Uhlschmid G. K., Neuenschwander P., Suter U. W. (1997). Development of degradable polyesterurethanes for medical applications: In vitro and in vivo evaluations.

[9] Borkenhagen M., Stoll R. C., Neuenschwander P., Suter U. W., Aebischer P. (1998). In vivo performance of a new biodegradable polyester urethane system used as a nerve guidance channel.

[10] Dong Z.-H., Zhang L., Li Y.-B., Zhou G., Lee S.-W. (2008). A guided bone regeneration membrane composed of hydroxyapatite and polyurethane.

[11] Selvakumar M., Srivastava P., Pawar H. S. (2016). On-Demand Guided Bone Regeneration with Microbial Protection of Ornamented SPU Scaffold with Bismuth-Doped Single Crystalline Hydroxyapatite: Augmentation and Cartilage Formation.

[12] Santerre J. P., Woodhouse K., Laroche G., Labow R. S. (2005). Understanding the biodegradation of polyurethanes: From classical implants to tissue engineering materials.

[13] Kavlock K. D., Pechar T. W., Hollinger J. O., Guelcher S. A., Goldstein A. S. (2007). Synthesis and characterization of segmented poly(esterurethane urea) elastomers for bone tissue engineering.

[14] Zdrahala R. J., Zdrahala I. J. (1999). Biomedical applications of polyurethanes: A review of past promises, present realities, and a vibrant future.

[15] Chandra R., Rustgi R. (1998). Biodegradable polymers.

[16] Zalipsky S. (1995). Chemistry of polyethylene glycol conjugates with biologically active molecules.

[17] Liechty W. B., Kryscio D. R., Slaughter B. V., Peppas N. A. (2010). Polymers for drug delivery systems.

[18] Park D., Wu W., Wang Y. (2011). A functionalizable reverse thermal gel based on a polyurethane/PEG block copolymer.

[19] Cherng J. Y., Hou T. Y., Shih M. F., Talsma H., Hennink W. E. (2013). Polyurethane-based drug delivery systems.

[20] Bhardwaj N., Kundu S. C. (2010). Electrospinning: a fascinating fiber fabrication technique.

[21] Cipitria A., Skelton A., Dargaville T. R., Dalton P. D., Hutmacher D. W. (2011). Design, fabrication and characterization of PCL electrospun scaffolds—a review.

[22] Zhang B. G. X., Quigley A. F., Myers D. E., Wallace G. G., Kapsa R. M. I., Choong P. F. M. (2014). Recent advances in nerve tissue engineering.

[23] Becker Edwin D. (1999).

[24] Nyquist, Richard A. (2001).

[25] Charles J. P., Jacqlynn B. (1993).

[26] Spindler R., Fréchet J. M. J. (1993). Synthesis and Characterization of Hyperbranched Polyurethanes Prepared from Blocked Isocyanate Monomers by Step-Growth Polymerization.

[27] Ingrisch S., Maier A., Wolfertstetter F., Winkelmann H., Alfred K., Weichmann J. (2002). Self-crosslinking polyurethane polymer hybrid dispersion.

[28] Hepburn C. (1992). Reaction rates, catalysis and surfactants.

[29] Singh H., Sharma T. P., Jain A. K. (2007). Reactivity of the raw materials and their effects on the structure and properties of rigid polyurethane foams.

[30] Eceiza A., Martin M. D., De La Caba K. (2008). Thermoplastic polyurethane elastomers based on polycarbonate diols with different soft segment molecular weight and chemical structure: Mechanical and thermal properties.

[31] Dotson N. A., Galvan R., Robert L. L., Tirrell M. (1995).

[32] Badami A. S., Kreke M. R., Thompson M. S., Riffle J. S., Goldstein A. S. (2006). Effect of fiber diameter on spreading, proliferation, and differentiation of osteoblastic cells on electrospun poly(lactic acid) substrates.

[33] Bashur C. A., Dahlgren L. A., Goldstein A. S. (2006). Effect of fiber diameter and orientation on fibroblast morphology and proliferation on electrospun poly(d,l-lactic-co-glycolic acid) meshes.

[34] Bottino M. C., Thomas V., Schmidt G. (2012). Recent advances in the development of GTR/GBR membranes for periodontal regeneration—a materials perspective.

[35] Wang C., Chien H.-S., Hsu C.-H., Wang Y.-C., Wang C.-T., Lu H.-A. (2007). Electrospinning of polyacrylonitrile solutions at elevated temperatures.

[36] Lee Y.-J., Park S.-J., Lee W.-K., Ko J. S., Kim H.-M. (2003). MG63 osteoblastic cell adhesion to the hydrophobic surface precoated with recombinant osteopontin fragments.

[37] Price N., Bendall S. P., Frondoza C., Jinnah R. H., Hungerford D. S. (1997). Human osteoblast-like cells (MG63) proliferate on a bioactive glass surface.

